# Utilizing cactus pear pruning residuals as sustainable growing media for containerized basil (*Ocimum basilicum* L:) cultivation

**DOI:** 10.1371/journal.pone.0334018

**Published:** 2025-10-10

**Authors:** Nicolò Auteri, Filippo Saiano, Riccardo Scalenghe, Alessandra Carrubba, Mauro Sarno

**Affiliations:** Dipartimento Scienze Agrarie, Alimentari e Forestali, Università degli studi di Palermo, Palermo, Italy; National Institute of Agricultural Research - INRA, MOROCCO

## Abstract

The increasing interest in sustainable and cost-effective options for containerized plant cultivation has driven research into the use of agricultural by-products and waste as alternative growing media. Cactus pear (*Opuntia ficus-indica* (L.) Mill.) pruning residuals, abundant in Mediterranean regions, represent a potential renewable resource. This study aimed to evaluate the suitability of cactus pear pruning residuals, enriched with calcium (Ca²⁺), iron (Fe²⁺ and Fe³⁺) ions, as a growing medium for basil (*Ocimum basilicum* L.) cultivation, with a focus on plant growth. From pots under greenhouse conditions, growth parameters (plant height, leaf area, number of leaves), chlorophyll content (SPAD), phosphorus availability in substrates (Olsen), and volatile compounds in leaves (HS-SPME coupled with GC-MS) were measured. Results suggest that incorporation of Ca- and Fe-enriched substrates significantly improved basil growth, with leading to better nutrient assimilation and higher growth metrics (plant height +23%; number of leaves +17%; leaf area +67%) compared to the untreated cactus pear substrate. Plants grown in Fe-enriched substrates exhibited increased plant height (+14%), leaf area (+48%), and number of leaves (+14%), along with improved phosphorus availability, compared to Ca^2+^ enrichments. The addition of 5% Fe^3+^ enriched cactus pear to the substrate resulted in increased plant height (+20%), number of leaves (+22%), and leaf area (+29%) compared to the control. Cactus pear pruning residuals, when enriched with Fe^3+^, show significant promise as a sustainable and cost-effective alternative to conventional growing media for basil cultivation, particularly in Mediterranean environments.

## 1. Introduction

Growing media (GMs) are a crucial component for supporting the growth and development of horticultural and ornamental containerized crops. Presently, conventional growing methods often rely on non-renewable resources; among these, peat has been long time considered a reference GM, but its use has been criticized both for economic reasons and the inherent negative impacts on the environment at a global scale [1]. To address this issue, there is a need for sustainable GMs that are made from renewable resources and can be recycled. The effectiveness of a GM depends on its ability to balance water and air, which is crucial for plant growth [2,3]. Many organic materials have been suggested for this purpose, and a special interest has been devoted to wastes and residuals, including woody residuals, animal manures, food industry wastes, and many others, mixed in various proportions and tested on several different crops [4–8]. Indeed, the disposal of waste is in many cases a serious issue and recycling for horticultural purposes can be a satisfactory option [9].

Using recycled organic GMs from pruning wastes can be a sustainable and cost-effective option for growing plants in greenhouse conditions. This organic matter from pruning waste can provide a suitable environment for plant growth, also improving soil structure, water-holding capacity, and fertility. Furthermore, as De Corato [10] noted, the production of high-quality composts from agricultural waste and by-products can provide a valuable source of eco-friendly organic molecules and beneficial microorganisms.

The literature suggests that basil (*Ocimum basilicum* L.), a fast-growing annual herb, is well-suited for greenhouse cultivation in pots packed with different GMs due to its quick germination and colonization abilities [11]. However, a proper choice of growing substrates is of paramount importance to achieve a high-quality product [12]. In some environments, the abundance of locally available agricultural waste makes it a promising and eco-sustainable option for GMs [13,14]. Hence, the possibility to add cactus pear pruning residuals -a cheap and largely available biomass in Mediterranean environments [15]- as partial or total substitute of peat could offer an eco-sustainable alternative for Mediterranean horticultural practices [16,17]. However, it is important to note that the addition of recycled materials can affect water and nutrient availability to crops. For instance, Bondì et al. [18] reported that cactus pear pruning residue can significantly influence soil bulk density and water retention.

Research has shown that pH, iron, and calcium influence the growth of basil cultivation in GMs [19,20]. This trial aims to evaluate whether cactus pear pruning waste can serve as a sustainable and cost-effective option for basil cultivation. To the best of our knowledge, this is the first attempt to convert un‑composted pruning biomass, enriched *in situ* with Ca^2+^/Fe^2+^/Fe^3+^, into a peat‑free potting medium for basil. This circular approach not only adds value to an abundant agricultural by‑product but also reduces reliance on peat, representing the key innovation of our work.

## 2. Materials and methods

### 2.1. Location and experimental design

The experiment was conducted between May and July 2022 in Palermo (38°06′27″ N 13°21′09″ E, 42 m a.s.l.). The local climate is temperate subtropical (CS; annual mean temperature >17°C; the mean temperature of the coldest month >10°C; 5 months with mean temperature >20°C; annual temperature range 13°C to l7°C) according to Koppen classification [21].

Cladodes from Opuntia ficus-indica (L.) Mill. were collected during the fall season in Roccamena (IT) (37°50'17"88 N, 13°9'20"16 E; 472 m a.s.l.).

Thirteen substrates (treatments) were prepared, each containing commercial potting soil (T) and increasing cactus pear (*Opuntia ficus-indica* (L.) Mill.) powder content (CP, added at 2.5%, 5%, 10% w/w), both untreated and differently endowed with Ca^2+^, Fe^2+^ or Fe^3+^ ions (Table 1). All prepared substrates were placed in plastic truncated cone pots having an upper diameter of 5.7 cm, a lower diameter of 3.6 cm, a height of 5.3 cm and a total volume of 91 cm^3^. Inside a greenhouse, five commercial basil seeds (*Ocimum basilicum* L. cv ‘Blumen’) were sown in each pot on May 20, 2022. Each treatment was replicated 5 times (Supplementary Information S1 Fig).

**Table 1:** Characterization of the substrates tested.

	ID treatment	% CP	Treatments	CP^(1)^ pre-treatment	pH	EC^(2)^ dS m^-1^	DBD^(3)^ g m^-3^	Ca^(4)^ content mg g^-1^	Fe^(4)^ content mg g^-1^	Initial P content (P_i_) µg pot^-1^
1	T_0_	0	T	–	7.6	3.530	0.36	5.0 ± 1.32	0.11 ± 0.05	0.38 ± 0.12
2	T_1_	2.5	T + CP	–	7.0	2.946	0.37	6.3 ± 1.11	0.12 ± 0.04	0.39 ± 0.06
3	T_1_	5	T + CP	–	7.6	2.795	0.39	7.6 ± 1.93	0.23 ± 0.06	0.88 ± 0.15
4	T_1_	10	T + CP	–	7.8	2.361	0.40	10.2 ± 1.74	0.44 ± 0.11	1.19 ± 0.07
5	T_2_	2.5	T + Ca-CP-P	CaCl_3_	7.6	2.580	0.36	9.4 ± 1.61	0.12 ± 0.04	0.37 ± 0.12
6	T_2_	5	T + Ca-CP-P	CaCl_3_	7.7	2.131	0.38	13.9 ± 1.12	0.23 ± 0.06	0.59 ± 0.09
7	T_2_	10	T + Ca-CP-P	CaCl_3_	7.7	1.805	0.40	22.8 ± 1.33	0.44 ± 0.11	1.48 ± 0.12
8	T_3_	2.5	T + Fe^2+ -^CP-P	FeSO_4_	7.0	1.964	0.35	6.3 ± 1.11	1.52 ± 1.27	0.37 ± 0.17
9	T_3_	5	T + Fe^2+ ^-CP-P	FeSO_4_	7.6	1.870	0.36	7.6 ± 1.93	3.05 ± 1.33	0.92 ± 0.16
10	T_3_	10	T + Fe^2+ ^-CP-P	FeSO_4_	7.4	1.774	0.38	10.2 ± 1.74	6.09 ± 1.42	1.72 ± 0.21
11	T_4_	2.5	T + Fe^3+ ^-CP-P	FeCl_3_	7.0	2.519	0.35	6.3 ± 1.11	2.58 ± 2.41	0.36 ± 0.13
12	T_4_	5	T + Fe^3+ ^-CP-P	FeCl_3_	7.5	1.876	0.38	7.6 ± 1.93	5.16 ± 2.24	0.40 ± 0.08
13	T_4_	10	T + Fe^3+ ^-CP-P	FeCl_3_	7.7	1.352	0.39	10.2 ± 1.74	10.31 ± 2.5	0.47 ± 0.28

(1)CP: untreated cactus pear powder; ^(2)^EC: electrical conductivity; ^(3)^DBD: dry bulk density. ^(4)^mg of Ca or Fe per gram of substrate

### 2.2. Substrates preparation

Commercial potting soil (T) was used (Universal Potting Soil Radicom^®^, VigorPlant Italia, L.L.C.), suitable for all gardens, vegetable gardens and terrace plants, with peat (a mix of fine white and brown types) making up as much as 65% of its composition.

Cactus pear powder (CP) was dissected, dried at 60°C for 72 hours and ground, bringing the particles to an average diameter between 250 µm and 2 mm. Then, the obtained material was treated with Ca^2+^, Fe^2+^ or Fe^3+^ ions, aiming to bridge and facilitate the chemical and physical interaction between the organic matrix and the phosphate anions. At this point, three separate materials were obtained, namely: Ca^2+^ - calcium loaded cactus pear powder (Ca-CP), Fe^2+^- ferrous iron loaded cactus pear powder (Fe^2+^-CP), Fe^3+^- ferric iron loaded cactus pear powder (Fe^3+^-CP), which were used to conduct the P adsorption batch experiments. Regardless of the received treatment (modified to Ca^2+^, Fe^2+^ or Fe^3+^ ions), cactus peer powder demonstrated the ability to remove P from phosphate solutions with varying percentages of removal. Thus, at the end of the batch experiment, we obtained three P-enriched starting materials (Ca-CP-P, Fe^2+^-CP-P, Fe^3+^-CP-P). The pots were prepared by mixing the potting soil (T) with both the untreated cactus pear biomass and the different types of biomasses, first treated with Ca or Fe and then enriched in P; three different biomass contents (2.5%, 5% and 10% w/w on total substrate) were supplied for each treatment. Table 1 shows the composition and the main characteristics (pH, bulk density, electrical conductivity, and initial Ca, Fe, and P contents with standard deviation) of the 13 individual growing substrates. Further details were previously published [17].

#### 2.2.1. Basil: Growth parameters.

Regardless of the used substrate, seed germination occurred between 1 and 14 days after sowing (DAS); by 14 DAS, the average germination rate had reached 54% with no significant differences observed between substrates, including the controls (binomial GLM; P > 0.05). To avoid competition for nutrients and light, the smallest and thinnest plantlets were removed, leaving only one plantlet for pot. At 20 DAS, all pots containing fully established plantlets were moved outdoors, in a location sheltered from wind and rain.

The experiment was considered over at 64 DAS. Hence, plants’ growth was monitored from 28 to 68 DAS, taking note of the following parameters: plantlets’ height, leaf area plant^-1^, chlorophyll content (SPAD), and the number of leaves plant^-1^. In total, five measurements of the above parameters were taken, at 28, 35, 45, 55, and 68 DAS.

The data about seedlings’ height, leaf area development, and the number of leaves per plant were acquired by taking photographs including the 5 replications of each treatment and a metric reference, further analyzed with the image analysis software Digimizer v. 4.6.1 (MedCalc Software, 2005–2016).

On 51 and 64 DAS, due to the increased size of basil leaves, SPAD measurements were taken in all treatments using the SPAD-502 meter (Minolta corporation, Ltd., Osaka, Japan). Being quick and non-invasive determinations, SPAD values are a reliable and widely used way to measure chlorophyll content in leaves and are often used as an indicator of plant health in horticultural research [6,22–24].

#### 2.2.2. Basil: Volatile compounds.

On air-dried and ground leaves of basil samples growth in the different substrates, headspace solid phase microextraction (HS-SPME) analyses were performed. Sample aliquots (about 250 mg) were placed in 20 mL glass vials sealed with a silicon septum and stored at 4°C until analysis. The SPME fiber (DVB/CAR/PDMS coated with divinylbenzene/carboxen/polydimethylsiloxane, 50 μm, Supelco), was conditioned for 2 h at 250°C in the gas chromatograph inlet. After 60 min of equilibration at 25°C, the SPME fiber was recovered and inserted into the injector port of the gas chromatograph, allowing for 2 min desorption at 250°C. Three replicates of each sample were analysed. A gas chromatographic instrument (Agilent 6890), with a Flame ionization detector (FID) and a mass selective (MS) detector (Agilent 5975c), was used with a Carbowax capillary column (30 m length, 0.25 mm internal diameter, and 0.25 μm film thickness from Supelco). Chromatographic conditions: injector in splitless mode at 250°C, carrier gas Helium, at 1 mL min^-1^ and an oven temperature program of a 5 min isotherm at 40°C, a linear temperature increase of 4°C min^-1^ to 200°C, held for 2 min. The FID was to 250°C while MS scan conditions were: source temperature 230°C, interface temperature 280°C, and mass scan range 33–350 amu. The NIST05 library was used for compound identification. The volatiles profile was described by comparisons of mass-spectra with high-quality materials and confirmed by comparisons of their retention indices (RI) with data in the available literature or co-injection of authentic standards available in the laboratory.

### 2.3. Substrates: Available phosphorus

In all substrates, available phosphorus (P) content was determined at the beginning (P_*i*_, 24 DAS) and at the end (P_f_, 64 DAS) of the experiment, to assess any eventual variation of P available to plants throughout their development. Available P content was assessed through the Olsen method [25]: 2 grams of samples were weighed, to which 40mL of an extractive solution (0.5 mol L^-1^) of sodium bicarbonate (pH 8.5) and 0.5 g of activated carbon was added. After shaking for 30 minutes, the samples were filtered with Whatman No. 42 paper, collecting the filtrate in 50 mL Falcon tubes, followed by spectrophotometric determination at 720 nm.

### 2.4. Statistical analysis

All treatments were submitted to analysis of variance (ANOVA), according to a randomized design with 5 replications. Prior to analysis, variance homogeneity was checked in all the investigated variables by means of the Levene’s test (p-values>>0.05). A General Linear Model (GLM; Y = f(x)) was adopted, in which the determinations on plants and substrates were the dependent variable (Y), whereas the experimental factors (measurement date in days after sowing – DAS, different P-enrichments, and mixing ratios) were the independent variables (X). The Tukey’s HSD test was run when significant differences (P ≤ 0.05) were observed among the treatments, and, to achieve a better insight of the effects of the most relevant groups of treatments, an Orthogonal Contrast (OC) analysis was performed within the factor “treatment” [26,27]. Although the OC technique allows performing a maximum number of independent comparisons (each with 1 DF) corresponding to the DF of the analysed factor (in our case, 12), our analysis was limited to the 6 more meaningful contrasts, *a priori* assigned in the planning phase of the experiment (Table 2). All statistical analyses were performed using the statistical package Minitab^®^ 17.1.0 (Minitab Inc., State College, PA, USA, 2013).

**Table 2:** Composition of the 6 planned orthogonal contrasts.

Id	Description	Group A	Group B
*C1*	Control *vs* all treatments	T_0_	T_1_, T_2_, T_3_, T_4_
*C2*	Natural CP *vs* treated CP	T_1_	T_2_, T_3_, T_4_
*C3*	CP + Ca *vs* CP + Fe^2 + ^, CP + Fe^3+^	T_2_	T_3_, T_4_
*C4*	CP + Fe^2 +^ *vs* CP + Fe^3+^	T_3_	T_4_
*C5*	CP_2.5_ *vs* CP_5_, CP_10_	T_2-2.5_, T_3-2.5_, T_4-2.5_	T_2-5_, T_3-5_, T_4-5,_ T_2-10,_ T_3-10,_ T_4-10_
*C6*	CP_5_ *vs* CP_10_	T_2-5_, T_3-5_, T_4-5_	T_2-10_, T_3-10_, T_4-10_

## 3. Results

### 3.1. Growth parameters measured on plants

Seed germination occurred within 1–14 DAS and averaged 54% by 14 DAS, with no statistically significant differences among any of the substrate treatments or the control (binomial GLM, P > 0.05).

**Fig 1:**
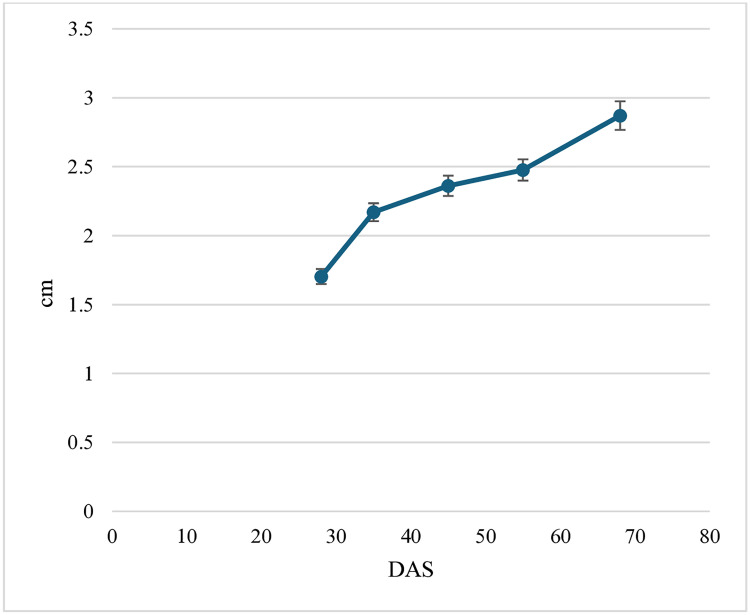
Average trend of basil plants height (cm) measured throughout the whole observation period (28 to 68 DAS). Each value is the average of 13 treatments (including control) x 5 replications. Vertical bars represent the standard deviation of each mean.

From the ANOVA conducted on the parameter “plant height” (Table 3), both factors “date” and “treatment” (but not their interaction) caused significant differences (p < 0.001) on plant height values. In the absence of a significant interaction, the mean values across “date” and “treatment” could be discussed separately. In the timespan from 28 to 68 DAS, this variable showed a regular increase (Fig 1).

**Table 3:** Results of the ANOVA on plantlets’ height values (cm).

Source	DF	F-value	P-value
Replication	4	4.145714	0.003
Date (D)	4	53.49247	<0.001
Treatment	12	10.75589	<0.001
*C1 (control vs all treatments)*	*1*	*0.395509*	0.530
*C2 (Natural CP vs treated CP)*	*1*	*43.79962*	<0.001
*C3 (CP + Ca vs CP + Fe*^*2 + *^*, CP + Fe*^*3+*^)	*1*	*17.02118*	<0.001
*C4 (CP + Fe*^*2 +*^* vs CP + Fe*^*3+*^)	*1*	*0.300913*	0.584
*C5 (CP*_*2.5*_ *vs CP*_*5,*_ *CP*_*10*_)	*1*	*2.279276*	0.132
*C6 (CP*_*5*_ *vs CP*_*10*_)	*1*	*0.895809*	0.345
Date × Treatment (err D)	48	0.801382	0.819
Error	229		
Total	297		

**Fig 2:**
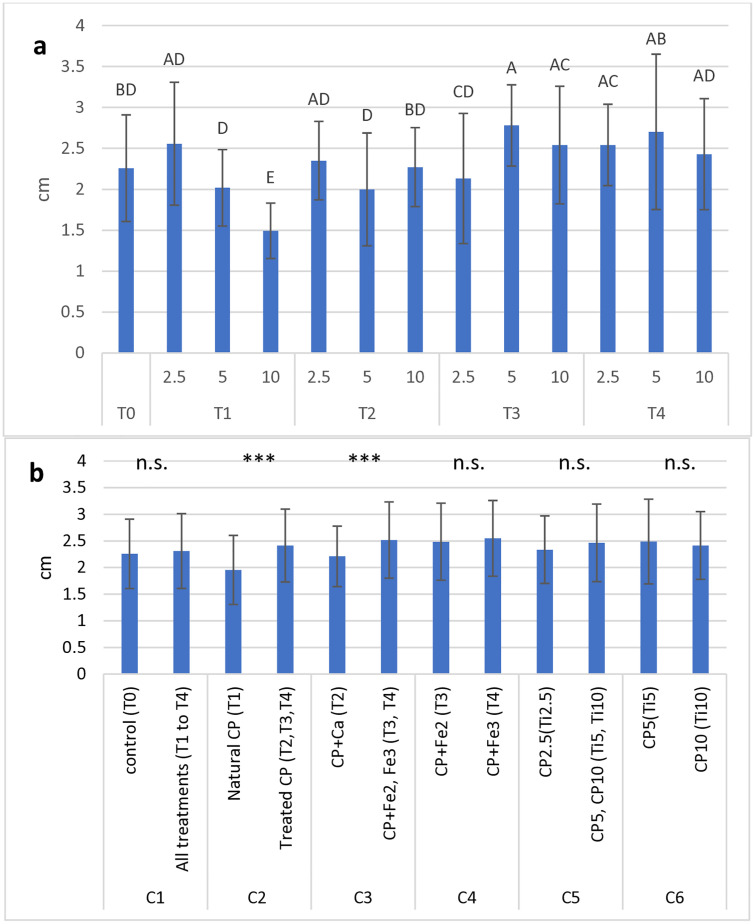
Mean values of basil plants height (cm) measured in 13 treatments (including control) (a) and in all groups tested for OC analysis (b). Each histogram represents the average of 5 observation times x 5 replications. Vertical bars represent the standard deviation of each mean. In (a), letters refer to the results of Tukey’s HSD test; means with the same letter (including not reported intermediates) are significantly not different at p ≤ 0.05. In (b), symbols above each contrast refer to the results of the OC analysis; n.s.: not significant; ***: significant at p ≤ 0.001.

Plant height (Fig 2a) spanned between the lowest value (less than 1.5 cm) in the T_1-10_ treatment (T containing 10% cactus pear) and the highest value (2.78 cm) in the T_3-5_ treatment (T + Fe^2+^ with 5% cactus pear). The addition of natural CP had a negative impact on plant heights, as demonstrated by the decreasing trend of values from the lowest (2.5%) to the highest (10%) CP percentage.

At the OC analysis (Fig 2b), highly statistical differences (p < 0.001) showed up between the natural and the treated cactus pear substrates (T_1_ *vs* T_2_, T_3_, T_4_), since the second group averaged much higher value (2.41 cm) than the first one (1.95 cm). Within the treated CP substrates, the addition of Ca (2.21 cm) allowed significantly lower height values than the addition of Fe (2.52 cm), with no difference between Fe^2^ and Fe^3^.

**Fig 3:**
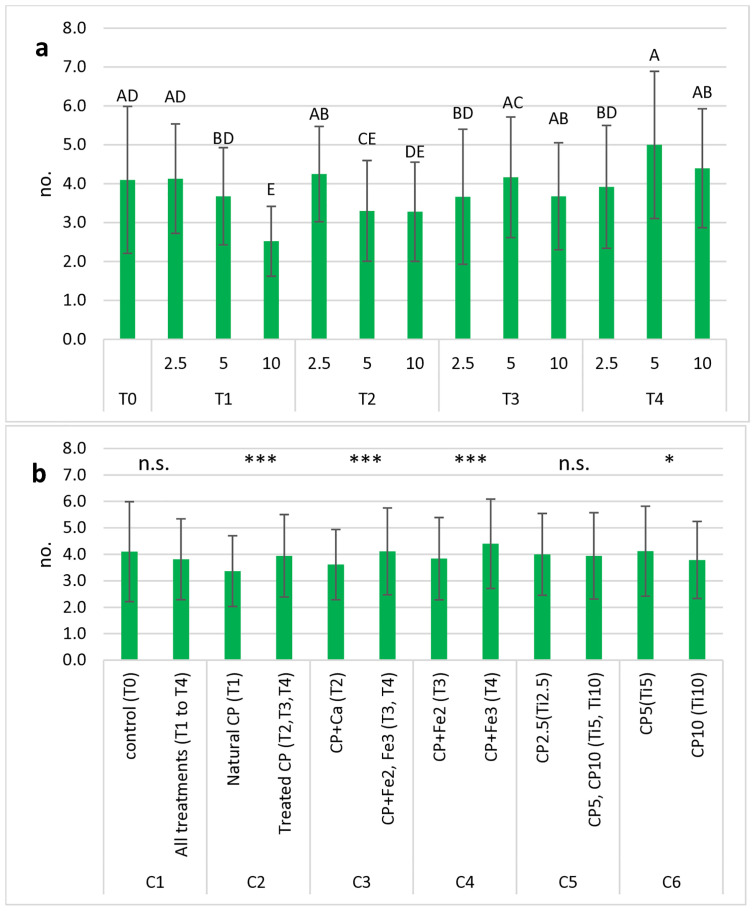
Mean values of number of leaves per plant in basil plants measured in 13 treatments (including control) (a) and in all groups tested for OC analysis (b). Each histogram represents the average of 5 observation times x 5 replications. Vertical bars represent the standard deviation of each mean. In (a), letters refer to the results of Tukey’s HSD test; means with the same letter (including not reported intermediates) are significantly not different at p ≤ 0.05. In (b), symbols above each contrast refer to the results of the OC analysis; n.s.: not significant; *: significant at p ≤ 0.05; ***: significant at p ≤ 0.001.

The ANOVA carried out on the number of leaves per plant (Table 4) showed the occurrence of highly significant differences (p < 0.001) due to both experimental factors “date” and “treatment”, whereas their interaction was not significant. As with the height of plantlets, the lowest number of leaves per plant could be detected in the T_1–10_ treatment (Fig 3a), whereas the highest mean was found in the T_4-5_ treatment. On average (Fig 3b), the group of treated cactus pear gave a higher number of leaves per plant than the “natural” cactus pear; the Fe treatments had a higher number of leaves than those treated with Ca. Furthermore, Fe^3+^ had significantly better outcome than Fe^2+^. Among cactus pear rates, 5% had a significantly higher number of leaves than 10%.

**Table 4:** Results of the ANOVA on the number of leaves plant^-1^ (n).

Source	DF	F-Value	P-Value
Replicates	4	8.060795	0.005
Date (D)	4	76.88315	<0.001
Treatments	12	9.587296	<0.001
*C1 (control vs all treatments)*	*1*	*1.09236*	0.297
*C2 (Natural CP vs treated CP)*	*1*	*22.7514*	<0.001
*C3 (CP + Ca vs CP + Fe*^*2 + *^*, CP + Fe*^*3+*^)	*1*	*12.47466*	<0.001
*C4 (CP + Fe*^*2 +*^* vs CP + Fe*^*3+*^)	*1*	*10.6115*	0.001
*C5 (CP*_*2.5*_ *vs CP*_*5,*_ *CP*_*10*_)	*1*	*0.39461*	0.531
*C6 (CP*_*5*_ *vs CP*_*10*_)	*1*	*5.24266*	0.023
Date × Treatment (err D)	48	1.384428	0.061
Error	229		
Total	297		

**Fig 4:**
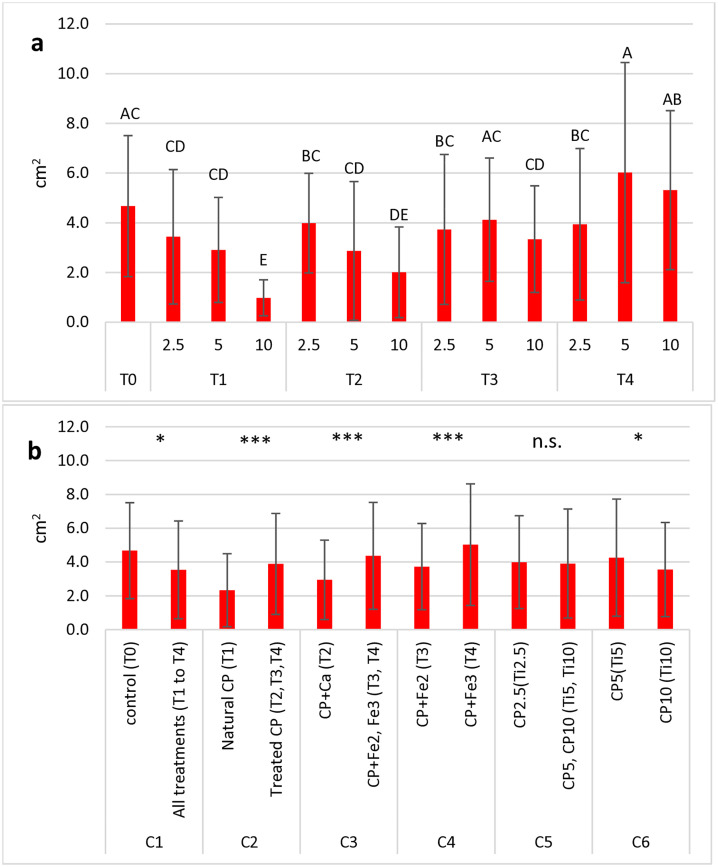
Mean values of the leaf area per plant (cm^2^) in basil plants measured in 13 treatments (including control) (a) and in all groups tested for OC analysis (b). Each histogram represents the average of 5 observation times x 5 replications. Vertical bars represent the standard deviation of each mean. In (a), letters refer to the results of Tukey’s HSD test; means with the same letter (including not reported intermediates) are significantly not different at p ≤ 0.05. In (b), symbols above each contrast refer to the results of the OC analysis; n.s.: not significant; *: significant at p ≤ 0.05; ***: significant at p ≤ 0.001.

The ANOVA conducted on the variable “leaf area per plant” (Table 5) enlightened that both factors “date” and “treatment” caused significant differences (p < 0.001), but no effect of their interaction was assessed. As shown (Fig 4a), the lowest leaf area was measured in the treatment T_1-10_, whereas the highest value was found in the treatment T_4-5_.

**Table 5:** Results of the ANOVA on the leaf area per plant (cm^2^).

Source	DF	F-Value	P-Value
Replicates	4	3.495381	0.009
Date (D)	4	61.82243	<0.001
Treatments	12	11.36677	<0.001
*C1 (control vs all treatments)*	*1*	*5.55001*	0.019
*C2 (Natural CP vs treated CP)*	*1*	*37.0357*	<0.001
*C3 (CP + Ca vs CP + Fe*^*2 + *^*, CP + Fe*^*3+*^)	*1*	*25.53389*	<0.001
*C4 (CP + Fe*^*2 +*^* vs CP + Fe*^*3+*^)	*1*	*14.8188*	<0.001
*C5 (CP*_*2.5*_ *vs CP*_*5,*_ *CP*_*10*_)	*1*	*0.20679*	0.650
*C6 (CP*_*5*_ *vs CP*_*10*_)	*1*	*6.30559*	0.013
Date × Treatment (err D)	48	1.1822	0.210
Error	229		
Total	297		

The OC analysis (Fig 4b) evidenced a wider leaf area in the control pots (T_0_) than in all cactus pear-treated substrates, and within them, treated cactus pear showed a higher leaf area than untreated cactus pear. Fe-enriched substrates, especially Fe^3^, had a higher leaf area than Ca. Among cactus pear rates, 5% had a slightly better performance than 10%.

**Fig 5:**
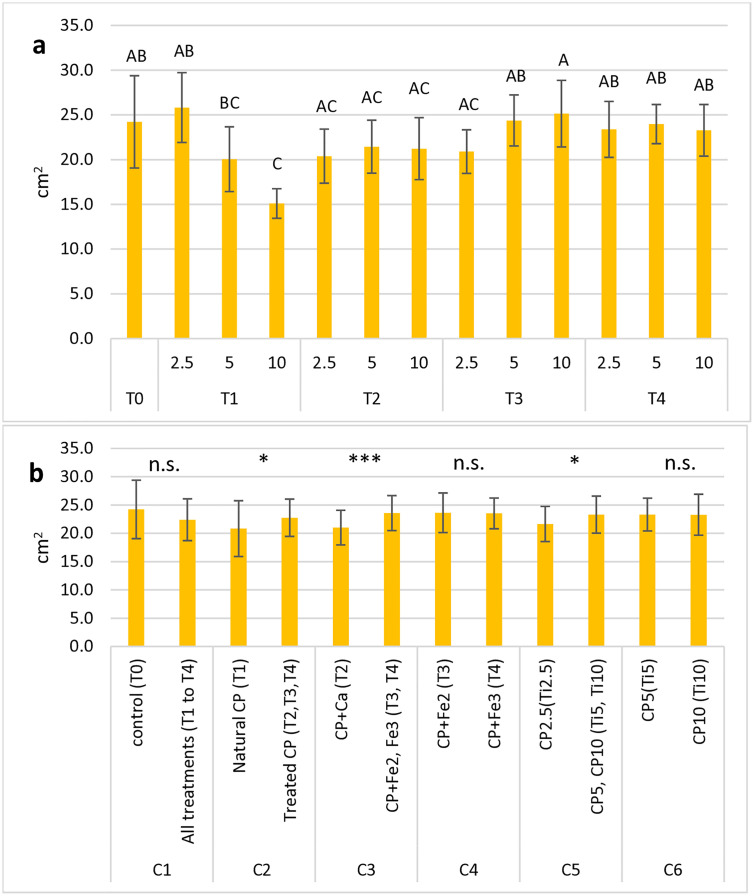
Mean values of the SPAD values in basil plants measured in 13 treatments (including control) (a) and in all groups tested for OC analysis (b). Each histogram represents the average of 5 observation times x 5 replications. Vertical bars represent the standard deviation of each mean. In (a), letters refer to the results of Tukey’s HSD test; means with the same letter (including not reported intermediates) are significantly not different at p ≤ 0.05. In (b), symbols above each contrast refer to the results of the OC analysis; n.s.: not significant; *: significant at p ≤ 0.05; ***: significant at p ≤ 0.001.

Finally, some differences were noted in SPAD measurements (Table 6). Although a difference (p < 0.05) could be noted at the ANOVA between the first and the second measurement date, the highest variability was because of treatment (p < 0.001). As previously assessed for the other variables, the lowest value was averaged in the T_1-10_ treatment (Fig 5a), whereas the others showed rather similar values, also like those reported by other researchers [28] on Italian basil leaves.

**Table 6:** Results of the ANOVA on the SPAD measurements.

Source	DF	F-Value	P-Value
Replicates	4	0.190076	0.943
Date (D)	1	7.541693	0.018
Treatment	12	3.583455	<0.001
*C1 (control vs all treatments)*	*1*	*2.46397*	0.121
*C2 (Natural CP vs treated CP)*	*1*	*5.22596*	0.025
*C3 (CP + Ca vs CP + Fe*^*2 + *^*, CP + Fe*^*3+*^)	*1*	*10.87099*	0.001
*C4 (CP + Fe*^*2 +*^* vs CP + Fe*^*3+*^)	*1*	*0.02015*	0.888
*C5 (CP*_*2.5*_ *vs CP*_*5,*_ *CP*_*10*_)	*1*	*4.8833*	0.030
*C6 (CP*_*5*_ *vs CP*_*10*_)	*1*	*0.01736*	0.896
Date × Treatment (err D)	12	0.842581	0.607
Error	75		
Total	104		

The observation of the differences between groups (Fig 5b) confirmed the occurrence of significant differences between the “natural” and the “treated” cactus pear, and between the cactus pear treated with Fe and that treated with Ca. Among cactus pear percentages, the lowest rate (2.5%) had significantly lower values than 5% and 10%.

### 3.2. Available P in the substrates

**Fig 6:**
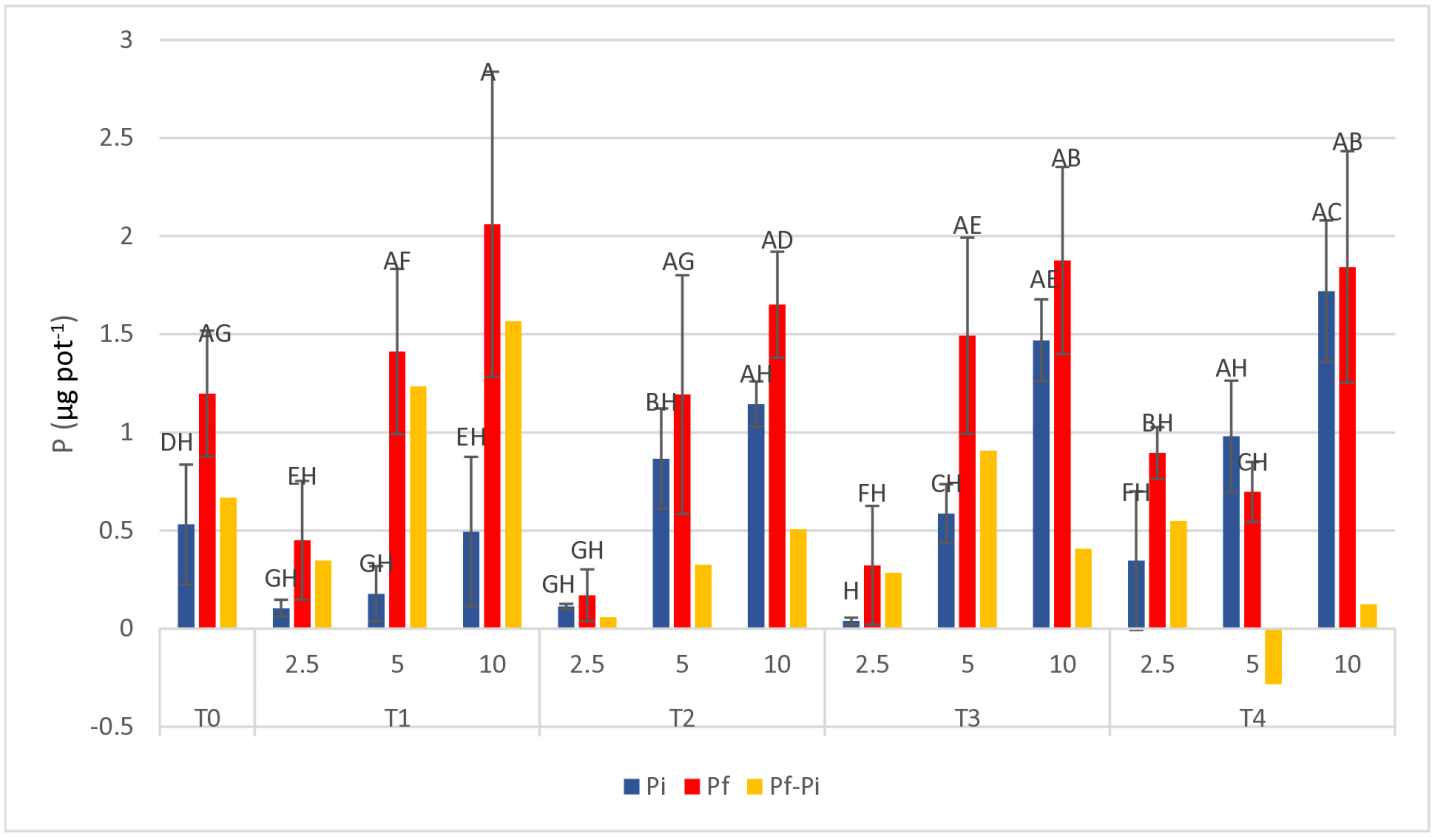
Initial (P_*i*_) and final (P_*f*_) available P content, and calculated P_*f*_-P_*i*_ difference, in the 12 tested substrates and in the control (T_0_). Each value is the average of 5 replications. Vertical bars represent the standard deviation of each mean.

Statistical analysis conducted on the initial and final values of available P content in the tested substrates showed highly significant differences due to both experimental factors (date and treatment), as well as to their interaction (p = 0.003). Hence, significant variations could be found between the two survey dates and among the different treatments, and treatments behaviour resulted different over time. The graph in Fig 6 brings evidence of a marked increase in time of available P in rather all treatments including the control. Furthermore, the comparison between the different CP rates for each treatment clearly evidences a higher level of available P with increasing the CP content within the substrate.

### 3.3. Volatile compounds in the basil leaves

No significant differences among treatments were detected in the composition of VOCs. In total, about 22 substances were found in all basil leaves that had been harvested for this investigation. The major volatile compounds were linalool (30.5–35.6%) and 1,8-cineole (9.6–12.5%) while other compounds with significant amounts were β-elemene (3.0–4.5%), valencene (0.9–2.7%), 2-pentanone (0.4–1.4%).

## 4. Discussion

The absence of germination differences confirms that Ca- or Fe-enriched cactus-pear substrates do not interfere with seed emergence, so subsequent treatment effects can be ascribed to post-emergence growth responses rather than differential establishment.

The descriptive analysis conducted on the leaf area of plantlets over time, grouping the information collected by CP treatment type, shows that the different CP content in the tested substrates influenced plant growth. Basil plants grown on the T_4-5_ substrate had an overall better response than the control (T_0_) in terms of height (+20.4%), leaf area (+28.7%) and number of leaves per plant (+21.9%), and a moderate SPAD value, lower only than plants grown on the T_3–5_ substrate. A similar response was found in the substrate T_4–10_, with the difference that the increase in the biomass content within that substrate slightly reduced all the variables considered, but recorded a significant increase in available P_f_.

Plants on the T_3–5_ treatment also reached a similar height value to those grown in the T_4–5_ substrate. However, the same trend was not exhibited by leaf area, which was lower than both the control and the T_4-5_. This behaviour was not repeated in the other substrates, which differed by type of treatment received (Ca, Fe^2+^ and/or Fe^3+^) or by CP biomass content (2.5, 5, and/or 10%). The positive effects on basil growth on T_4–5_, T_4–10_, or T_3-5_ could therefore be attributed to the presence of Fe in the chemical composition of the biomass, which promotes nutrient assimilation [29].

Iron, a critical element, is involved in various cellular processes in plants, such as chlorophyll synthesis, photosynthesis, and respiration [30]. Despite its abundance in soil, Fe^3+^ in its oxidized, insoluble form prevails under aerobic conditions, making it inaccessible to plants [30]. To overcome the limited availability of Fe, higher plants have developed strategies to acquire Fe from the rhizosphere, with non-graminaceous plants reducing soil pH and converting Fe^3+^ to soluble Fe^2+^ via Fe^3+^-chelate reductase and Fe^2+^ transport [31]. However, Fe^3+^ is a more stable and effective form of iron for plant growth than Fe^2+^. Fe^2+^ is readily oxidized to Fe^3+^, but is not as easily absorbed by plant roots, while Fe^3+^ is more easily absorbed and less likely to form insoluble compounds in the soil, making it more available to plants over a longer period [32]. In our experiment, the addition of Fe^3+^-loaded cactus pear to the growing media helped the plants ensure access to the soluble iron they needed for growth.

Caballero et al. [33] observed that chlorophyll meter readings (SPAD) were significantly related to pH of drainage water, with lower SPAD readings and dry matter yields at higher pH values but did not find significant correlations with iron additions to different growing media in their study about gerber. Unlike N availability, which was found to affect SPAD values due to an increase in the plant’s chlorophyll content [34], P availability did not affect SPAD measurements. Our results align with those of Hauck et al. [35], who demonstrated significant variability in the plant availability of P from different recycled sources, underscoring the importance of source-specific evaluation.

Certain scholars investigating the genus *Ocimum* have observed notable distinctions in the chromatographic spectrum of plants exposed to various treatments, particularly when it comes to light conditions. For instance, Chutimanukul et al. [36] found higher proportions of methyl eugenol, caryophyllene, and total phenylpropanoids, but total monoterpenoids and diterpenoids were not detected. Gurkan and Hayaloglu [37] found that the drying process led to the formation or increased presence of specific compounds: the dominant volatile compound in dried basil leaves samples were 1,8-cineole and linalool and a minor amount of 2-pentanone, β-elemene and valencene. In our experiment, the use of distinct growing substrates did not lead to relevant differences in the composition of volatile substances in basil leaves.

## 5. Conclusions

The different cactus pear powder content in the substrates influenced basil growth, with only the T_4-5_ treatment (T + Fe^3+^-CP with 5% cactus pear) showing a significantly higher plant height compared to the control. While the addition of cactus pear led to a slightly reduced leaf area compared to the control, the number of leaves per plant increased over time in all treatments, except for the T_1-10_ treatment, which experienced a slight reduction.

Throughout the experiment, in almost all treatments the available phosphorus (P) content in the substrates showed a significant increase between the initial and final survey dates, as well as among the different treatments. The ancillary P recovery obtained using cactus pear biomass not only offers a sustainable method for nutrient recycling, but also addresses the dual challenge of waste management and soil fertility improvement [18,38].

Basil plants grown on substrates with Fe^3+^-loaded cactus pear exhibited an overall better response compared to the control group. These plants showed improvements in height, leaf area, and the number of leaves per plant, while maintaining a moderate SPAD value—lower only than the plants grown on the substrate with the highest biomass content. The positive effects on basil growth were likely due to the presence of Fe in the biomass, which facilitated nutrient assimilation.

Although additional research is needed, also extending the surveys to a longer period (including plant’s flowering time), these preliminary results suggest that cactus pear residuals hold great promise as an alternative substrate for containerized cultivation of basil. Agricultural by-products have emerged as a promising option for sustainable horticultural practices, potentially replacing peat in certain situations. These by-products are readily available in local communities and come at minimal cost, making them an attractive alternative substrate.

## Supporting information

S1 Fig Acquisition of leaf height (a) and leaf area per pot (b). (DOCX)
