# Supplementary material for: Utilizing cactus pear pruning residuals as sustainable growing media for containerized basil (Ocimum basilicum L:) cultivation
Source: PLoS One. 2025 Oct 10;20(10):e0334018. doi: 10.1371/journal.pone.0334018 (PMC12513590; doi:10.1371/journal.pone.0334018)
Supplement: S1 Fig — (DOCX) [file pone.0334018.s001.docx]

**Supplementary Information**

**SI1 Growth parameters measured on basil plants**

Basil seeds were sown on May 20, 2022 (5 seeds pot^-1^) in plastic truncated cone pots having an upper diameter of 5.7 cm, a lower diameter of 3.6 cm, a height of 5.3 cm and a total volume of 80 cm^3^. Regardless of the used substrate, seed germination was recorded between 1 and 14 days after sowing (DAS; Figure 1), and at 20 DAS, all pots containing fully established plantlets were positioned outdoors, sheltered from wind and rain.

The experiment was considered over at 64 DAS. Hence, plants’ growth was monitored from 24 to 64 DAS, taking note of the following parameters: plantlets’ height, leaf area pot^-1^, SPAD values, and number of leaves plant^-1^. In total, five measurements of the above parameters were taken, at 31, 41, 51, and 64 DAS.

The data about seedlings’ height, leaf area development in each pot, and the number of leaves per plant were acquired by taking photographs including the 5 replications of each treatment and a metric reference, further analyzed with the image analysis software Digimizer v. 4.6.1 (MedCalc Software, 2005-2016) (Figure 1). When more than 1 plant was present in each pot, the average value of all individuals was considered.

**Figure SI1.** Acquisition of leaf height (a) and leaf area per pot (b)
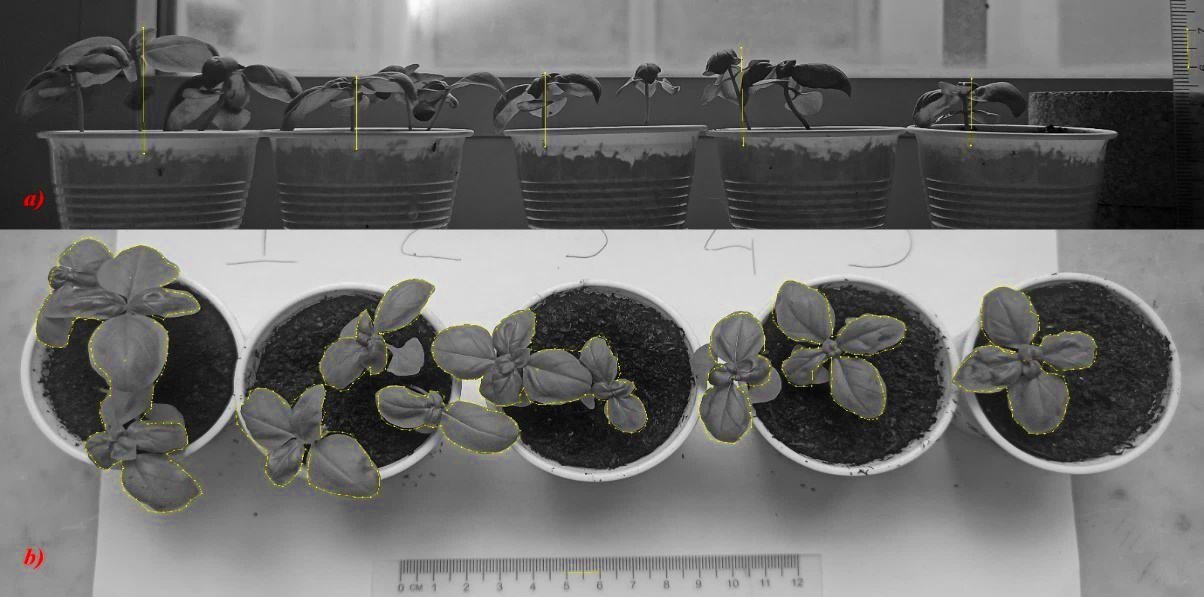


On July 14 and July 27, due to the increased size of basil leaves, SPAD measurements were taken in all treatments by means of the SPAD-502 meter (Minolta corporation, Ltd., Osaka, Japan). Being quick and noninvasive determinations, SPAD values are widely used in horticultural research as a key indicator of plant health and represent a reliable measurement of chlorophyll content in leaves (Markwell et al., 1995; Uddling et al., 2007).

***References***

Markwell, J., Osterman, J.C., Mitchell, J.L., (1995). Calibration of the Minolta SPAD-502 leaf chlorophyll meter. Photosynth. Res 46, 467–472. https://doi.org/10.1007/BF00032301.

Uddling, J., Gelang-Alfredsson, J., Piikki, K., Pleijel, H., (2007). Evaluating the relationship between leaf chlorophyll concentration and SPAD-502 chlorophyll meter readings. Photosyntheses Res. 91, 37–46. https://doi.org/10.1007/s11120-006-9077-5.
